# Left atrial volume assessment in atrial fibrillation using multimodality imaging: a comparison of echocardiography, invasive three dimensional CARTO and cardiac magnetic resonance imaging

**DOI:** 10.1186/1532-429X-14-S1-P214

**Published:** 2012-02-01

**Authors:** Mark Rabbat, David Wilber, Kevin M Thomas, Anoop Agrawal, Thriveni Sanagala, Mushabbar A Syed

**Affiliations:** 1 Heart and Vascular Institute, Loyola University Chicago, Maywood, IL, USA

## Background

Atrial fibrillation (AF) is the most common cardiac arrhythmia. Accurate assessment of left atrial (LA) volumes is imperative, as LA size is a strong predictor of successful ablation and cardiovascular events. Cardiac magnetic resonance imaging (CMR) using the multiple slice method (MSM) is the current gold standard for volumetric analysis; however, it is time consuming. Thus, we sought to determine whether LA volume assessment using the more rapid area length (AL) method on transthoracic echocardiography (TTE), AL method on CMR, and invasive measurement by 3D-CARTO electrophysiologic mapping correlated with CMR MSM.

## Methods

We prospectively studied 141 consecutive patients with AF from July 1, 2010 to July 31, 2011 prior to AF ablation (Table 1). CMR images were acquired on a 3T scanner (Siemens Trio) by SSFP using a short axis stack, horizontal long-axis and vertical long-axis of the LA to measure LA volumes by MSM and biplane AL method. TTE apical 2- and 4-chamber views were obtained during LA end-diastole and areas were used to calculate LA volumes using the biplane AL method. LA volumes during ablation were measured by electrophysiologic mapping using 3D-CARTO technique. Atrial volumetric measurements were compared using intra-class correlation coefficients (ICC), Pearson’s correlation and Bland-Altman plots. CMR MSM was used as the reference standard.

**Table 1:** Baseline Patient Characteristics at the Time of Ablation

Parameter	Patients (n=141)
Age	59 +/- 10
Male	101 (72)
BMI	29 +/- 6
Type of AF	
Paroxysmal AF	77 (55)
Persistent AF	48 (34)
Permanent AF	9 (6)
AF duration (months)	64 +/- 77
Number of failed AAD	1.1 +/- 0.92
History of Structural Heart Disease (SHD)	55 (39)
Diabetes	22 (15.6)
Hypertension	78 (55)
CHADS-2 score of >1	30 (21)

Data are expressed as mean +/- standard deviation (SD) or number (%) of patients. AAD = antiarrhythmic drug.

## Results

Out of 141 patients, 138 underwent CMR, 92 underwent TTE and 122 underwent 3D-CARTO. Mean LA volumes estimated using the MRI-AL method were significantly higher compared to MSM (125+/-38 ml vs 114+/-34 , p <0.005). Mean LA volumes using 3D-CARTO and TTE-AL were significantly lower compared to MSM (106+/-33 ml vs 112+/-31, p <0.005, and 94+/-27 ml vs 117+/-35, p <0.005 respectively). MRI-AL method overestimated LA volumes by 11.6% whereas 3D-CARTO and TTE-AL underestimated LA volumes by 4.9% and 11.7% respectively (Table 2). MRI-AL and 3D-CARTO correlated well with MSM (ICC=0.8 and 0.77 respectively). However, TTE-AL had poor correlation with MSM (ICC=0.48). Bland-Altman plots confirmed the above findings. There were no significant gender differences in LA volumetric assessment between imaging modalities.

**Table 2:** Left Atrial Volume Measurements Comparing Echocardiography, Invasive 3D-CARTO and CMR

Imaging Modality	Absolute Difference	Percent Difference	ICC	Pearson Correlation	R2
MRI-AL vs MSM	11.48 +/- 20.45*	11.6 +/- 19.95*	0.80*	0.84*	0.71*
3D-CARTO vs MSM	-6.42 +/- 20.80*	-4.92 +/- 17.00*	0.77*	0.79*	0.62*
TTE-AL vs MSM	-23.84 +/- 26.68*	-17.44 +/- 21.08*	0.48*	0.65*	0.42*

Atrial volume measurements are in mL. Values are presented as means and SD. MSM=multiple slice method. AL=area length method. TTE=transthoracic echocardiography. ICC=intraclass correlation coefficient. R2=coefficient of determination. *= p-value <0.005.

## Conclusions

In AF patients undergoing catheter ablation, TTE-AL significantly underestimates LA volumes and has poor correlation with CMR MSM. MRI-AL significantly overestimates LA volumes while 3D-CARTO significantly underestimates LA volumes, but both correlate well with CMR MSM. As an alternative to CMR MSM, MRI-AL should be the preferred technique to measure LA volumes in AF patients undergoing catheter ablation, as it is non-invasive, rapid, and correlates well with CMR MSM.

## Funding

None.

